# Older Adults’ Perceptions of Disposition Decisions from the Emergency Department

**DOI:** 10.1093/geroni/igab046.2283

**Published:** 2021-12-17

**Authors:** Karen Valcheff, Hoffart Nancy

**Affiliations:** 1 University of North Carolina -Chapel Hill, Greensboro, North Carolina, United States; 2 University of Notrht Carolina, Greensboro, Greensboro, North Carolina, United States

## Abstract

Patient-centered care strives to improve older adult outcomes from the emergency department (ED). Appropriate disposition decisions from the ED for older adults are becoming increasingly complex and challenging. The purpose of this study was to explore the perceptions of older adults as to their disposition from the emergency department, the decision making process, and their engagement in that process. The Three-Talk Shared Decision Making (SDM) model guided the study. A qualitative approach was used to interview seven older adults two days after being treated in the ED. Transcribed data were thematically analyzed using MAXQDA to identify codes, patterns, and themes. Analysis revealed that the Three-Talk SDM model was not being used. Participants identified only one option regarding their disposition from the ED and perceived they had little voice in decision making. They reported a variety of emotional reactions, feelings of helplessness and empathy regarding the decision making process. Three factors that participants perceived as vital to them before making a disposition decision were safety, pain relief, and a definitive diagnosis. The findings of this small sample are clinically meaningful. These older adults wanted to be heard regarding their treatment and disposition decisions. Findings indicate the need for provider education about the use of a model such as the Three -Talk SDM. Further research is needed to look at both the older adult and provider’s perception of the ED disposition decision. Additional strategies and skills are warranted to enhance shared decision making in the ED with the growing aging population.

